# Reductions in inpatient fluoroquinolone use and postdischarge *Clostridioides difficile* infection (CDI) from a systemwide antimicrobial stewardship intervention

**DOI:** 10.1017/ash.2021.197

**Published:** 2021-10-22

**Authors:** K. Ashley Jones, Udodirim N. Onwubiko, Julianne Kubes, Benjamin Albrecht, Kristen Paciullo, Jessica Howard-Anderson, Sujit Suchindran, Ronald Trible, Jesse T. Jacob, Sarah H. Yi, Dana Goodenough, Scott K. Fridkin, Mary Elizabeth Sexton, Zanthia Wiley

**Affiliations:** 1 Department of Pharmacy, Emory Healthcare, Atlanta, Georgia; 2 Department of Epidemiology, Rollins School of Public Health, Emory University, Atlanta, Georgia; 3 Office of Quality, Emory Healthcare, Atlanta, Georgia; 4 Division of Infectious Diseases, Department of Medicine, Emory University School of Medicine, Atlanta, Georgia; 5 Georgia Emerging Infections Program, Atlanta, Georgia; 6 Division of Healthcare Quality Promotion, National Center for Infectious Diseases, Centers for Disease Control and Prevention, Atlanta, Georgia; 7 Foundation for Atlanta Veterans’ Education & Research, Decatur, Georgia; 8 Atlanta Veterans’ Affairs Medical Center, Decatur, Georgia

**Keywords:** fluoroquinolone antimicrobial stewardship, post-discharge C. difficile infection, hospital-onset C. difficile infection, clinical decision support

## Abstract

**Objective:**

To determine the impact of an inpatient stewardship intervention targeting fluoroquinolone use on inpatient and postdischarge *Clostridioides difficile* infection (CDI).

**Design:**

We used an interrupted time series study design to evaluate the rate of hospital-onset CDI (HO-CDI), postdischarge CDI (PD-CDI) within 12 weeks, and inpatient fluoroquinolone use from 2 years prior to 1 year after a stewardship intervention.

**Setting:**

An academic healthcare system with 4 hospitals.

**Patients:**

All inpatients hospitalized between January 2017 and September 2020, excluding those discharged from locations caring for oncology, bone marrow transplant, or solid-organ transplant patients.

**Intervention:**

Introduction of electronic order sets designed to reduce inpatient fluoroquinolone prescribing.

**Results:**

Among 163,117 admissions, there were 683 cases of HO-CDI and 1,104 cases of PD-CDI. In the context of a 2% month-to-month decline starting in the preintervention period (*P* < .01), we observed a reduction in fluoroquinolone days of therapy per 1,000 patient days of 21% after the intervention (level change, *P* < .05). HO-CDI rates were stable throughout the study period. In contrast, we also detected a change in the trend of PD-CDI rates from a stable monthly rate in the preintervention period to a monthly decrease of 2.5% in the postintervention period (*P* < .01).

**Conclusions:**

Our systemwide intervention reduced inpatient fluoroquinolone use immediately, but not HO-CDI. However, a downward trend in PD-CDI occurred. Relying on outcome measures limited to the inpatient setting may not reflect the full impact of inpatient stewardship efforts.

*Clostridioides difficile* infection (CDI) typically occurs after a patient is exposed to antibiotics, with subsequent alteration in gut microbial flora.^1^ The Centers for Disease Control and Prevention (CDC) considers CDI an “urgent threat” to human health; it is now the most common pathogen detected among healthcare-associated infections (HAIs) in hospitalized patients.^2,3^ However, most CDIs occur outside the hospital, usually within a few months of hospital discharge.^4^ When including both inpatient and outpatient infections, ∼500,000 CDI cases and an associated 29,000 deaths occur each year in the United States.^4,5^

As a result, CDI prevention is a critical focus, and both decreasing unnecessary antibiotic use and shifting antibiotic prescribing to classes with a lower CDI risk have potential benefits.^6–8^ Antimicrobial stewardship interventions focused on reducing the use of high-risk antibiotics are a core component of CDI prevention efforts.^6,9,10^ Although nearly all antibiotics have been implicated in CDI, certain classes, such as fluoroquinolones (FQs), are associated with a higher risk of disease.^7,11,12^ Stewardship programs that implement coordinated interventions have reduced FQ prescribing in the inpatient setting,^13–16^ but ASPs have rarely resulted in reductions in hospital-onset CDI (HO-CDI) in nonoutbreak settings.^14,15^ Alterations in the gut microbiome secondary to antibiotic exposure are thought to last for weeks to months^11^; thus, it is possible that community cases of CDI may be affected by reductions in inpatient prescribing, though this hypothesis has not been well studied.

We implemented a healthcare systemwide electronic medical record (EMR) intervention aimed at decreasing FQ use in the inpatient setting. We sought to measure the impact of this intervention not only on inpatient FQ utilization and HO-CDI rates but also on incidence of CDI occurring within 12 weeks of discharge (ie, postdischarge CDI or PD-CDI).

## Methods

### Study setting and population

The study was conducted at 4 geographically separate acute-care hospitals in a single healthcare system in the Atlanta metropolitan area. The acute-care hospitals range in size from 100 to 550 beds, comprising ∼1,600 beds in total. Hospital A is a mixed academic and community tertiary-care hospital with ∼500 beds. Hospital B is an academic tertiary-care hospital with ∼550 beds. Hospitals C and D are community hospitals with ∼400 and ∼100 beds, respectively.

Data on antibiotic utilization and CDI rates for all 4 hospitals were collected for a 23-month baseline period (September 2017 until July 2019) and a 13-month postintervention period (September 2019 through September 2020) after implementation of an electronic medical record (EMR) intervention in August 2019. All inpatients were included, with the exception of patients admitted to locations dedicated to oncology, bone marrow transplant, and solid-organ transplant services because the intervention did not target the use of FQs for prophylaxis (eg, for neutropenia), which was a common indication for use in those locations. The hospitals’ institutional review boards approved the study under expedited review with a waiver of consent. This study included key reporting elements outlined in the Strengthening the Reporting of Observational Studies in Epidemiology (STROBE) reporting guideline.

### Stewardship intervention

In August 2019, all 4 hospitals implemented electronic order sets targeting a reduction in FQ use. The formulary FQs levofloxacin and ciprofloxacin were removed as standalone orders such that inpatient orders for FQs could only be placed through order sets with built-in clinical decision support. The levofloxacin and ciprofloxacin order sets listed the drugs’ FDA warnings and redirected prescribers to use syndrome-based order sets developed by the healthcare system’s antimicrobial stewardship program. For example, the levofloxacin order contained links to syndrome-based order sets for treatment of community-acquired pneumonia (CAP), urinary tract infection (UTI), and chronic obstructive pulmonary disease (COPD) exacerbations. These syndromic order sets included alternatives to FQs consistent with professional society recommendations and our institutional antibiograms, built-in stop dates for the shortest effective duration of therapy, and guidance for antimicrobial selection in patients with β-lactam allergies. If a provider did continue to place an order for levofloxacin or ciprofloxacin, they were required to select the indication from a prepopulated list.

### Data collection and definitions

Using an existing clinical data warehouse, we obtained antibiotic administration data and CDI laboratory test results, as well as patient-encounter data to allow for calculation of the Medicare Severity Diagnosis Related Group case-mix index (MS-DRG CMI).

Antibiotic use data were generated based on barcode medication administration that had been validated internally. Each antibiotic administration was attributed to the patient care location where it was given, and those locations were grouped by National Healthcare Safety Network (NHSN) defined locations: hematology–oncology locations (excluded from this analysis), critical care locations, and non–critical care locations (wards). Days of therapy (DOT) for each NHSN location were obtained for both FQs and other broad-spectrum antibiotics. FQ use was defined as either oral or intravenous administration of ciprofloxacin or levofloxacin. Broad-spectrum hospital-onset (BS-HO) antibiotics (in our system consisted of carbapenems, piperacillin/tazobactam, and third- and fourth-generation cephalosporins) were defined using the NHSN definition^17^ to have a second antibiotic use rate as a nonequivalent dependent variable. We considered BS-HO antibiotic group as a nonequivalent dependent variable because the intervention was not designed to switch from an FQ to the BS-HO group.

For detection of HO-CDI and PD-CDI, diarrheal stool specimens that tested positive in any of our healthcare system laboratories using polymerase chain reaction (PCR) tests (Xpert *C. difficile* PCR assay, Cepheid, Sunnyvale, CA) were included. Positive tests were labeled as HO-CDI if they occurred during an inpatient stay after the third day of admission.^18^ HO-CDI encounters were attributed to the location of the patient on the date that the diarrheal specimen was collected. Encounters were labeled as PD-CDI if testing was positive between the date of discharge and 12 weeks after discharge.

To improve the sensitivity of detecting PD-CDI, all eligible encounters were also reviewed by the CDC-funded Georgia Emerging Infections Program (GA EIP). GA EIP conducts active population-based CDI surveillance in the 8-county metropolitan Atlanta area (2019 population, 4.16 million). Eligible patient encounters from the 4-hospital system were linked to incident positive (no positive in previous 8 weeks) CDI laboratory tests from September 2017 through December 2020 to identify PD-CDI from any laboratory serving the 8-county metropolitan Atlanta area. However, to avoid misclassifying HO-CDI occurring in other hospitals as a PD-CDI from 1 of our 4 hospitals, only episodes identified at ambulatory and referral laboratories (in addition to laboratories within our health system) counted toward the calculation of 12-week PD-CDI episodes.

### Statistical analysis

Monthly antibiotic use rates (DOT per 1,000 patient days), HO-CDI incidence rates (per 1,000 patient days), and PD-CDI rates (per 100 patient discharges) were calculated and described for each intervention period using median and interquartile range for counts or median and bootstrapped 95% confidence intervals for rates. Metrics for individual hospitals as well as pooled metrics for system-level estimates were calculated.

Segmented negative binomial regression models allowing for both level and trend changes were used to estimate the impact of the intervention on each outcome. Each model included 3 terms: a continuous variable representing the study month, a binary “intervention” variable indicating preintervention status versus postintervention status, and a continuous variable representing the postintervention month (effectively an interaction between the study month and intervention variables). The 2 primary measures of interest from these models were change in level between the pre- and postintervention periods and change in rate trend between the 2 periods.

Since the intervention was implemented mid-August 2019, data were censored from August 2019 in all models to avoid misclassification of August data into either pre- or postintervention periods. Durbin Watson and Breusch-Godfrey tests were used to assess residual autocorrelation in the segmented regression models. When significant autocorrelation was detected by both tests (*P* < .05), robust standard errors were generated using a sandwich estimator to produce conservative estimates of uncertainty. All analyses were performed at the system level (using aggregated monthly data from all 4 hospitals) as well as the individual hospital level (stratified analysis). For system-level segmented regression analysis, generalized estimating equations models with a negative binomial link function and first-order autoregressive correlation structure were used to account for clustering by facility. Stratified analyses explored variations in impact to each hospital facility.

In March 2020, the midpoint of the postintervention period, the healthcare system canceled all elective surgery and procedures through early May 2020 in response to the COVID-19 pandemic. We examined the potential influence of the COVID-19 pandemic on the level and trend changes in the outcomes through 2 sensitivity analyses: first by adjusting for MS-DRG CMI and second by modeling the first month of COVID-19 admissions as a distinct change point in the interrupted time series analysis.^19–21^ All data analyses were conducted in R version 4.0.2 statistical software (R Foundation for Statistical Computing, Vienna, Austria) and SAS version 9.4 software (SAS Institute, Cary, NC).

## Results

### Antibiotic use

Overall, the numbers of patients discharged from the respective hospitals during the study period were as follows: hospital A (n = 36,757), hospital B (n = 51,134), hospital C (n = 45,223), and hospital D (n = 18,943). Distributions of pooled antibiotic use (DOT) in the preintervention period (September 2017–July 2019) and postintervention period (August 2019–September 2020) across all 4 facilities are shown in Table 1. The pooled median monthly rate of FQ use was 32.3 DOT per 1,000 patient days in the preintervention period and 16.4 DOT per 1,000 patient days in the postintervention period. Median use of BS-HO antibiotics was 171.1 DOT per 1,000 patient days prior to intervention and 192.7 DOT per 1,000 patient days after the intervention. An average drop in FQ use and an increase in BS-HO antibiotic use were also observed each of the 4 acute-care facilities (Supplementary Table S1).

**Table 1.** Pooled Total and Median Monthly Antibiotic (Fluoroquinolone and Broad Spectrum) Days of Therapy and *C. difficile* Infections (Hospital-Onset and After Discharge) in 4 Acute-Care Facilities in Atlanta, Georgia (September 2017–September 2020)

Variable	Preintervention Period (n = 23 months)			Postintervention Period (n = 13 months)		
	Total	Median Monthly Count (IQR)	Median Monthly Rate^a^ (95% CI^b^)	Total	Median Monthly Count (IQR)	Median Monthly Rate^a^ (95% CI^b^)
Fluoroquinolone DOT	21,153	928 (821.0–1026.5)	32.3 (29.0–35.7)	7,038	488.5 (413.3–568.8)	16.4 (15.3–18.2)
BS-HO DOT	111,686	4,797 (4,548.0–5,121.0)	171.1 (166.1–174)	77,559	5,694 (5,342.3–5,895.3)	192.7 (184.8–196.5)
Hospital-onset CDI	439	18 (16.5–22.5)	0.7 (0.6–0.8)	244	18 (14.0–20.0)	0.6 (0.5–0.7)
12-week postdischarge CDI^c^	734	30 (27.0–39.5)	0.7 (0.7–0.9)	370	24.5 (21.3–30.5)	0.6 (0.6–0.8)

Notes: IQR, interquqrtile range; CI, confidence interval; HO, hospital onset; BS-HO, broad-spectrum hospital-onset antibiotics were defined using the National Healthcare Safety Network (NHSN) definition; DOT, days of therapy; CDI, *Clostridiodes difficile* infection.

a Per 1,000 person days (fluoroquinolone DOT, BS-HO DOT and HO CDI); per 100 patient discharges (12-week postdischarge CDI)

b Bootstrapped 95% confidence intervals for pooled median outcome rates.

c CDI occurring in a patient in the outpatient setting within 12 weeks after discharge, processed by either healthcare-system laboratory (n = 634 preintervention, n = 334 postintervention) or any non–hospital-based laboratory in the 8-county metropolitan area catchment of the Georgia Emerging Infections Program (n = 59 preintervention, n = 36 postintervention).

Interrupted time series analysis of pooled data showed a baseline trend of 2.2% decline in FQ prescribing per month (*P* = .001) that continued in the postintervention period (Table 2). We detected a 21.3% decrease in the rate of FQ prescribing in the immediate postintervention period (level change rate ratio [RR], 0.787; 95% CI, 0.621–0.996; *P* = .047). We detected no level or trend changes detected in BS-HO prescribing rates.

**Table 2.** Changes in Fluoroquinolone and Broad-Spectrum (BSHO) Antibiotic Use and CDI (Hospital-Onset and 12 Weeks Postdischarge) in a System of 4 Acute-Care Facilities in Atlanta, Georgia (September 2017–September 2020)

Variable	Rate Ratio	95% CI		*P* Value
		Upper	Lower	
**Fluoroquinolone use**				
Baseline trend^a^	0.978	0.966	0.991	.001
Level change after intervention^b^	0.787	0.621	0.996	.047
Trend change after intervention^c^	0.998	0.988	1.008	.662
Postintervention trend^d^	0.976	0.969	0.983	.000
**Broad-spectrum antibiotics (NHSN BS-HO) use**				
Baseline trend^a^	1.002	0.996	1.007	.530
Level change after intervention^b^	1.053	0.937	1.185	.386
Trend change after intervention^c^	1.001	0.998	1.005	.492
Postintervention trend^d^	1.003	0.996	1.010	.373
**Hospital-onset CDI**				
Baseline trend^a^	1.001	0.988	1.014	.925
Level change after intervention^b^	0.898	0.657	1.226	.497
Trend change after intervention^c^	1.001	0.956	1.047	.984
Postintervention trend^d^	1.001	0.969	1.035	.949
**12-week postdischarge CDI**				
Baseline trend^a^	1.007	0.997	1.018	.182
Level change after intervention^b^	0.967	0.893	1.046	.400
Trend change after intervention^c^	0.968	0.947	0.989	.003
Postintervention trend^d^	0.975	0.962	0.988	<.001

Note. CDI, *Clostridioides difficile* infection; NHSN BS-HO, National Healthcare Safety Network broad-spectrum hospital-onset (BS-HO); CI, confident interval.

a Baseline month-to-month trend in rate, ie, exp(β1).

b Level change associated with intervention, ie, exp(β2).

c Change in month-to-month trend in the postintervention versus the preintervention period, ie, exp(β3).

d Month-to-month trend in the postintervention period only, ie, exp(β1+β3).

Each of the 4 acute-care facilities showed decreased FQ use in response to the intervention, with variations in the magnitude of level change (Supplementary Table S2).

### C. difficile *infections*

Across all 4 facilities over 37 months, there were 683 HO-CDIs and 1,104 PD-CDIs: 1,009 were identified from the primary healthcare system, and an additional 95 were identified in ambulatory settings throughout metropolitan Atlanta via GA EIP data (Table 1).

**Fig. 1.**
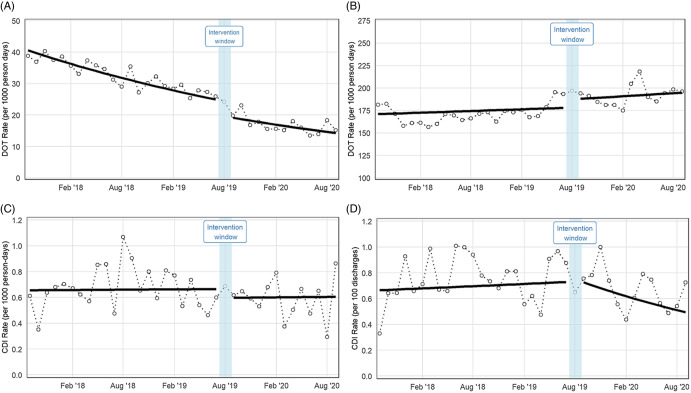
Antimicrobial utilization (fluoroquinolones, A; NHSN defined broad spectrum hospital-onset agents, B) and *C. difficile* infection rates (hospital onset, C; 12 weeks after discharge) before and after a fluoroquinolone reduction stewardship intervention across 4 acute-care hospitals, September 2017–September 2020.

The intervention was not associated with a change in the level or trend of HO-CDIs (Table 2). The monthly trend of 12-week PD-CDI rates changed between the pre- and postintervention periods (trend change RR, 0.968; 95% CI, 0.947–0.989; *P* = .003) from a stable monthly rate in the preintervention period to a monthly decrease of 2.5% in the postintervention period (Fig. 1).

Several sensitivity analyses were performed to confirm the impact of the intervention on PD-CDI. In the models using the pooled, systemwide data, MS-DRG CMI, and COVID-19 admissions were accounted for to adjust for changes since the start of the pandemic. The start of the COVID-19 pandemic coincided with an uptick in the monthly MS-DRG CMI across the 4-hospital system (Supplementary Fig. S1). The models incorporating monthly MS-DRG CMI produced a similar level change in FQ use and similar trend change as the respective primary analyses (Supplementary Fig. S2 and Table S3). The models incorporating a distinct change point for COVID-19 onset did not reveal level or trend changes associated with March 2020.

## Discussion

We have described a large healthcare system stewardship initiative that used an electronic order set with built-in decision support to decrease FQ inpatient prescribing. The intervention resulted in an immediate and significant decrease in inpatient FQ use beyond a pre-existing downward trend. Reaching all inpatient prescribers for successful implementation of antimicrobial stewardship interventions is challenging, especially across large healthcare systems staffed by both academic and community providers. Provider education and implementation of institutional guidelines require significant coordination and continuous effort.^22^ We leveraged the EMR by developing built-in decision support for antibiotic selection and duration, allowing for real-time guidance for prescribing FQs without an ongoing need for personnel resources or didactic education.

Although we did not see an associated impact on HO-CDI rates, we did detect a significant change from an upward to a downward trend of CDI incidence among recently discharged patients, from a stable rate to a steady and rapidly decreasing rate (−2.5% per month). This finding may reflect the fact that quantifying the impact of stewardship interventions on infection outcomes can be challenging because metrics related to HAI like HO-CDI are often affected by multimodal prevention efforts. For example, in this healthcare system, multidisciplinary interventions over several years have aimed to achieve low rates of HO-CDI, which may have obscured any measurable impact from a single additional intervention targeting FQ use. By expanding our evaluation of CDI incidence to include onset after discharge, we allowed more time to identify CDI attributed to the treatments provided during hospital stay that disrupted patients’ microbiomes.

In addition, we augmented our case ascertainment to include specimens submitted from ambulatory settings outside the healthcare system (detected by GA EIP surveillance), which strengthens our confidence that we were observing a true decrease in infections. Although this approach improved the accuracy of our case finding, very similar results with a significant decrease in PD-CDI were obtained when limiting case finding solely to our healthcare-system laboratories. This similarity suggests that other researchers could replicate these methods in sufficiently large healthcare networks.

This study had several limitations. We focused on an intervention solely related to FQ use, and other antibiotics also have great potential to disrupt the microbiome and lead to CDI.^7,11,12,23–25^ We also depended on retrospective case identification. FQ use at some hospitals was fairly low to start, and we saw a concomitant increase in BS-HO antibiotic utilization, either of which could have obscured an impact of decreased FQ utilization on HO-CDI rates. The most important limitation of our study was the lack a contemporaneous control group; however, we did analyze a nonequivalent dependent exposure, the NHSN-defined BS-HO group of antibiotics. Despite a stable level of use of these antibiotics, the observed decrease in the PD-CDI outcome, which we believe was a result of the intervention, was still observed on the PD-CDI outcome. Unfortunately, an unmeasured factor in the pathway of inpatient stewardship affecting PD-CDI is FQ prescribed at time of discharge; we were unable to measure the impact of our intervention on this activity.^17^ Additionally, our enhanced case finding for PD-CDI occurring outside the healthcare system and included only cases diagnosed in the ambulatory setting; we were unable to ensure that persons hospitalized outside our system at the time of diagnosis did not have HO-CDI. This inclusion maximized construct validity at the cost of reducing sensitivity of the metric. Our findings could be explained by less testing in the ambulatory setting after the intervention; however, this trend would have started before the COVID-19 pandemic. We attempted to account for the potential impact of COVID-19 by conducting sensitivity analyses, which yielded no differences in the main results.^19–21^

Finally, it was not possible to account for a multifactorial approach to reduce HO-CDI within our healthcare system, which included use of enhanced contact precautions, a dedicated program to improve hand hygiene, and diagnostic stewardship for *C. difficile* testing. Systemwide efforts targeting these areas were implemented early in the study period (2016–2017) and were maintained throughout the study period. These interventions likely had a much larger impact on HO-CDI rates than our stewardship intervention.^26–28^ However, they would not explain the change in PD-CDI observed after the stewardship intervention that occurred late in the study period, so that is likely the most critical finding.

Our systemwide intervention using electronic order sets with built-in decision support reduced inpatient FQ use by 21% beyond an ongoing drop in month-to-month FQ use that preceded the intervention. The intervention did not significantly reduce HO-CDI, but it significantly decreased 12 weeks PD-CDI. Our study adds to current literature supporting the effectiveness of decision support built into the EMR. Additionally, our study demonstrates that inpatient antimicrobial stewardship affects more than hospital metrics alone and can also affect patient health after discharge. Relying on outcome measures limited to inpatient settings may not reflect the full impact of inpatient stewardship efforts, and incorporating postdischarge outcomes, such as CDI, should be more widely considered.

## Supplementary material

For supplementary material accompanying this paper visit https://doi.org/10.1017/ash.2021.197. click here to view supplementary material
