# Supplementary material for: Reductions in inpatient fluoroquinolone use and postdischarge Clostridioides difficile infection (CDI) from a systemwide antimicrobial stewardship intervention
Source: Antimicrob Steward Healthc Epidemiol. 2021 Oct 22;1(1):e32. doi: 10.1017/ash.2021.197 (PMC9495417; doi:10.1017/ash.2021.197)
Supplement: Supplementary file 1 [file S2732494X21001972sup001.docx]

Supplementary Materials: Table S1. Facility-specific distribution of antibiotic (fluoroquinolone and broad-spectrum) days of therapy and *C. difficile* infections (hospital-onset and post-discharge) in four acute-care facilities in Atlanta, Georgia (September 2017 – September 2020).

|  | **Pre-Intervention period (n=23 months)** | | |  | **Post-Intervention period (n=13 months)** | | |  |
| --- | --- | --- | --- | --- | --- | --- | --- | --- |
|  | **Total Count** | **Median Monthly Count (IQR)^a^** | **Median Monthly Rate^b^ (95% CI**^c^**)** |  | **Total Count** | **Median Monthly Count (IQR)^a^** | **Median Monthly Rate^b^ (95% CI**^c^**)** |  |
| **Facility A:** |  |  |  |  |  |  |  |  |
| Fluoroquinolone DOT^e^ | 8,013 | 377 (265.0 - 409.0) | 35.6 (29.8 – 38.3) |  | 2,725 | 188.5 (150.5 - 234.0) | 21.4 (17.6 - 26.4) |  |
| BS-HO DOT^e^ | 46,638 | 2,006 (1952.5 - 2144.5) | 206.0 (196.1 - 209.0) |  | 28,282 | 2,100 (1979.8 - 2160.8) | 226.7 (218.7 - 236.1) |  |
| Hospital-Onset CDI^f^ | 187 | 8 (6.0 - 9.5) | 0.8 (0.6 – 0.9) |  | 107 | 8.5 (5.8 - 9.0) | 0.9 (0.7 - 1.0) |  |
| 12-week Post-discharge CDI^f^ | 194 | 8 (6.0 - 10.0) | 0.7 (0.6 - 0.8) |  | 63 | 4.5 (3.0 - 5.8) | 0.5 (0.4 - 0.6) |  |
| **Facility B:** |  |  |  |  |  |  |  |  |
| Fluoroquinolone DOT^e^ | 6,164 | 275 (252.0 - 297.0) | 31.0 (27.6 - 33.0) |  | 1,959 | 135 (114.3 - 144.8) | 13.5 (11.8 – 16.0) |  |
| BS-HO DOT^e^ | 28,715 | 1,240 (1151.0 - 1369.0) | 137.5 (133.3 - 140.6) |  | 21,276 | 1,566 (1417.0 - 1637.3) | 153.5 (148.6 – 163.0) |  |
| Hospital-Onset CDI^f^ | 165 | 7 (5.5 - 8.0) | 0.8 (0.7 - 0.9) |  | 77 | 5 (4.3 - 7.0) | 0.5 (0.4 - 0.6) |  |
| 12-week Post-discharge CDI^f^ | 250 | 11 (8.0 – 13.5) | 0.7 (0.6 - 0.9) |  | 120 | 8.5 (6.0 - 9.8) | 0.6 (0.5 - 0.7) |  |
| **Facility C:** |  |  |  |  |  |  |  |  |
| Fluoroquinolone DOT^e^ | 5,185 | 210 (202.0 - 250.5) | 31.9 (27.8 – 35.3) |  | 1,827 | 125 (99.8 - 176.8) | 15.9 (12.1 - 20.9) |  |
| BS-HO DOT^e^ | 25,932 | 1,102 (895.5 - 1325.5) | 166.7 (152.1- 170.1) |  | 21,264 | 1,520 (1422.0 - 1623.5) | 195.2 (185.3 - 205.2) |  |
| Hospital-Onset CDI^f^ | 55 | 3 (1.5 - 3.0) | 0.4 (0.3- 0.5) |  | 53 | 4 (3.0 - 4.8) | 0.5 (0.3 - 0.7) |  |
| 12-week Post-discharge CDI^f^ | 211 | 9 (7.0 - 12.0) | 0.7 (0.6- 0.8) |  | 138 | 9 (7.0 - 9.8) | 0.7 (0.5 - 0.8) |  |
| **Facility D:** |  |  |  |  |  |  |  |  |
| Fluoroquinolone DOT^e^ | 1,791 | 75 (55.0 - 92.0) | 29.6 (24.1 – 35.2) |  | 527 | 34.5 (28.3 - 45.8) | 14.0 (11.5 – 20.1) |  |
| BS-HO DOT^e^ | 10,401 | 448 (406.0 - 493.5) | 178.8 (167.8 – 189.1) |  | 6,737 | 484 (453.5 - 517.3) | 195.0 (185.7 – 206.3) |  |
| Hospital-Onset CDI^f^ | 32 | 1 (1.0 - 2.0) | 0.4 (0.4 - 0.7) |  | 7 | 0 (0.0 - 1.0) | 0.0 (0.0 - 0.4) |  |
| 12-week Post-discharge CDI^f^ | 79 | 4 (2.0 – 5.0) | 0.6 (0.4 - 0.8) |  | 49 | 3 (1.0 - 6.0) | 0.6 (0.2 - 1.2) |  |

^a^IQR – Interquartile Range

^b^Per 1000 person-days (Fluoroquinolone DOT, BSHO DOT and Hospital-Onset CDI); Per 100 patient discharges (12-week post-discharge CDI)

^c^Bootstrapped 95% Confidence Intervals for median rates

^e^DOT – Days of Therapy

^f^CDI-*Clostridiodes difficile* infections

Supplementary Materials: Table S2. Facility-specific changes in fluoroquinolone and broad-spectrum (BSHO) antibiotics use and *C. difficile* Infections (hospital-onset and 12-week post-discharge) in four acute-care facilities in Atlanta, Georgia (September 2017 – September 2020).

|  | **Fluoroquinolone Use** | |  | **BS-HO antibiotics** | |  | **Hospital-onset CDI** | |  | **12-week post-discharge CDI** | |
| --- | --- | --- | --- | --- | --- | --- | --- | --- | --- | --- | --- |
|  | **Rate Ratio (95% CI)** | **P value** |  | **Rate Ratio (95% CI)** | **P value** |  | **Rate Ratio (95% CI)** | **P value** |  | **Rate Ratio (95% CI)** | **P value** |
| **Facility A^e^** |  |  |  |  |  |  |  |  |  |  |  |
| Baseline trend^a^ | 0.980 (0.973 - 0.987) | <0.001 |  | 1.007 (1.001 - 1.012) | 0.013 |  | 1.018 (0.996 - 1.041) | 0.105 |  | 0.992 (0.971 - 1.014) | 0.480 |
| Level change^b^ | 0.962 (0.766 - 1.207) | 0.780 |  | 1.034 (0.952 - 1.122) | 0.429 |  | 1.034 (0.642 - 1.669) | 0.889 |  | 0.874 (0.485 - 1.574) | 0.653 |
| Trend change^c^ | 0.990 (0.966 - 1.015) | 0.736 |  | 0.993 (0.986 - 1.000) | 0.038 |  | 0.955 (0.903 - 1.011) | 0.114 |  | 0.990 (0.922 - 1.063) | 0.777 |
| Post-intervention trend^d^ | 0.970 (0.950 - 0.991) | 0.006 |  | 0.999 (0.990 - 1.009) | 0.859 |  | 0.973 (0.924 - 1.025) | 0.301 |  | 0.982 (0.918 - 1.051) | 0.599 |
| **Facility B** |  |  |  |  |  |  |  |  |  |  |  |
| Baseline trend^a^ | 0.985 (0.975 - 0.996) | 0.009 |  | 1.003 (0.996 - 1.009) | 0.401 |  | 0.997 (0.973 - 1.023) | 0.841 |  | 1.001 (0.985 - 1.024) | 0.656 |
| Level change^b^ | 0.565 (0.436 - 0.731) | <0.001 |  | 1.015 (0.919 - 1.122) | 0.763 |  | 0.684 (0.372 - 1.258) | 0.222 |  | 0.906 (0.577 - 1.423) | 0.669 |
| Trend change^c^ | 1.009 (0.980 - 1.039) | 0.546 |  | 1.008 (0.999 - 1.016) | 0.081 |  | 1.007 (0.939 - 1.080) | 0.846 |  | 0.970 (0.919 - 1.024) | 0.271 |
| Post-intervention trend^d^ | 0.994 (0.968 - 1.022) | 0.686 |  | 1.007 (0.992 - 1.023) | 0.375 |  | 1.004 (0.941 - 1.073) | 0.895 |  | 0.974 (0.927 - 1.025) | 0.314 |
| **Facility C** |  |  |  |  |  |  |  |  |  |  |  |
| Baseline trend^a^ | 0.989 (0.978 - 1.000) | 0.043 |  | 1.009 (1.000 - 1.018) | 0.061 |  | 1.009 (1.000 - 1.018) | 0.061 |  | 1.008 (0.986 - 1.031) | 0.481 |
| Level change^b^ | 0.670 (0.520 - 0.864) | 0.002 |  | 1.077 (0.978 - 1.186) | 0.131 |  | 1.077 (0.978 - 1.186) | 0.131 |  | 1.120 (0.706 – 1.775) | 0.631 |
| Trend change^c^ | 0.983 (0.955 - 1.013) | 0.262 |  | 0.995 (0.983 - 1.007) | 0.400 |  | 0.995 (0.983 - 1.007) | 0.400 |  | 0.972 (0.921 - 1.026) | 0.306 |
| Post-intervention trend^d^ | 0.972 (0.947 - 0.999) | 0.043 |  | 1.003 (0.989 - 1.018) | 0.641 |  | 1.003 (0.989 - 1.018) | 0.641 |  | 0.980 (0.933 - 1.030) | 0.420 |
| **Facility D** |  |  |  |  |  |  |  |  |  |  |  |
| Baseline trend^a^ | 0.956 (0.939 - 0.974) | <0.001 |  | 0.996 (0.989 - 1.003) | 0.238 |  | 0.987 (0.937 - 1.039) | 0.619 |  | 1.044 (1.008 - 1.080) | 0.015 |
| Level change^b^ | 0.996 (0.649 - 1.528) | 0.984 |  | 1.104 (0.940 - 1.297) | 0.228 |  | 0.595 (0.111 - 3.190) | 0.545 |  | 1.023 (0.516 - 2.025) | 0.949 |
| Trend change^c^ | 1.013 (0.964 - 1.064) | 0.610 |  | 1.009 (0.991 - 1.028) | 0.311 |  | 0.950 (0.760 - 1.188) | 0.653 |  | 0.892 (0.815 - 0.975) | 0.012 |
| Post-intervention trend^d^ | 0.968 (0.925 - 1.014) | 0.173 |  | 1.005 (0.989 - 1.022) | 0.549 |  | 0.937 (0.755 - 1.165) | 0.561 |  | 0.931 (0.857 - 1.011) | 0.087 |

^a^Baseline month-to-month trend in rate i.e. exp(β1)

^b^Step change associated with intervention i.e. exp(β2)

^c^Change in month-to-month trend in the post-intervention versus the pre-intervention period i.e. exp(β3)

^d^Month-to-month trend in the post-intervention period only i.e. exp(β1+β3)

^e^Sandwich estimators used to generate robust standard errors for confidence intervals

Supplementary Materials: Figure S1: Pooled Case Mix Index and COVID-19 diagnoses over time (September 2017-September 2020)


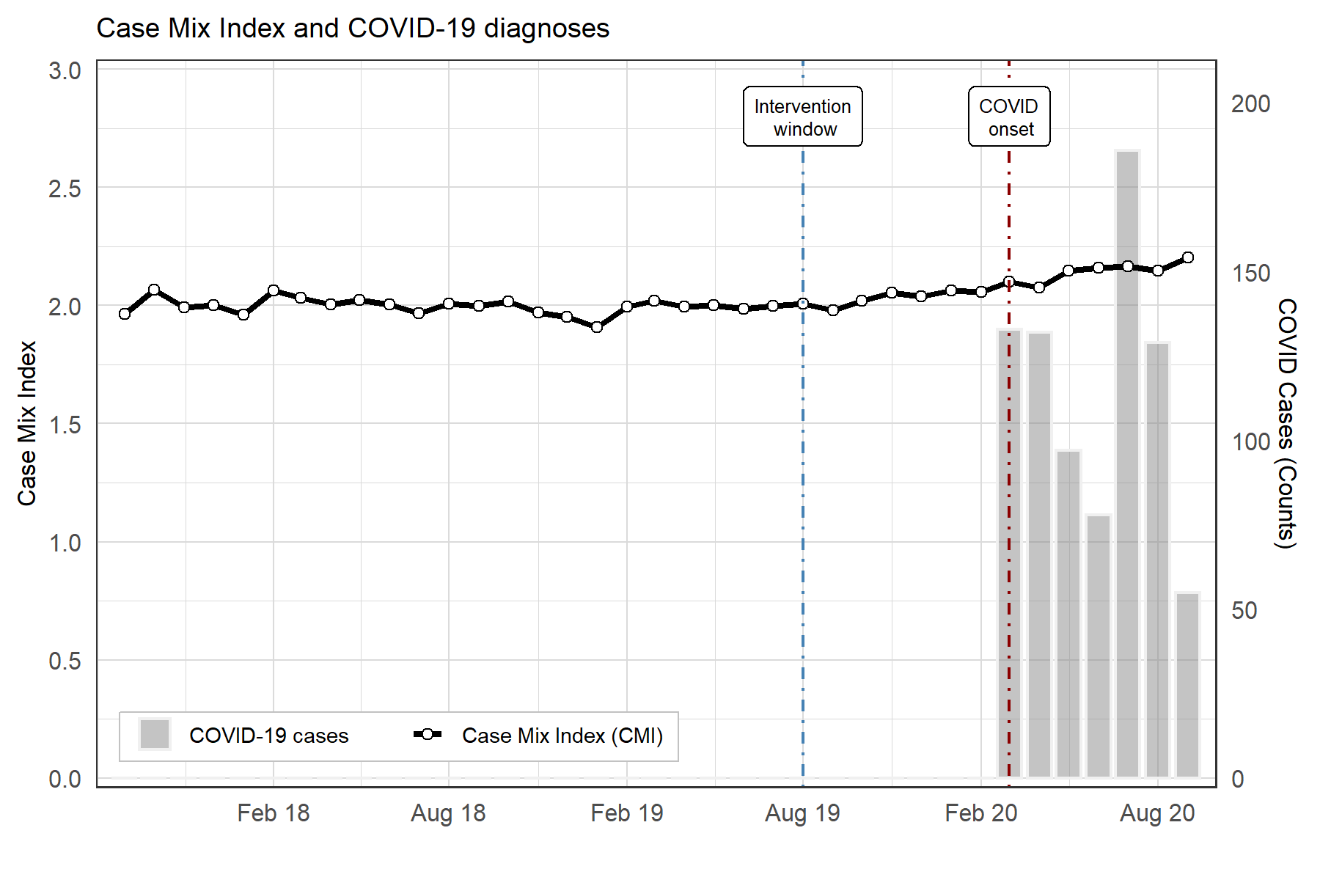


Supplementary Materials: Figure S2. Sensitivity Analysis of impact of intervention on fluoroquinolone antibiotic use (A, C) and 12-week post discharge *C. difficile* infections (B, D) at four acute-care facilities, with adjustment for MS-DRG CMI (A, B) or a COVID-time point (D, C), September 2017 – September 2020


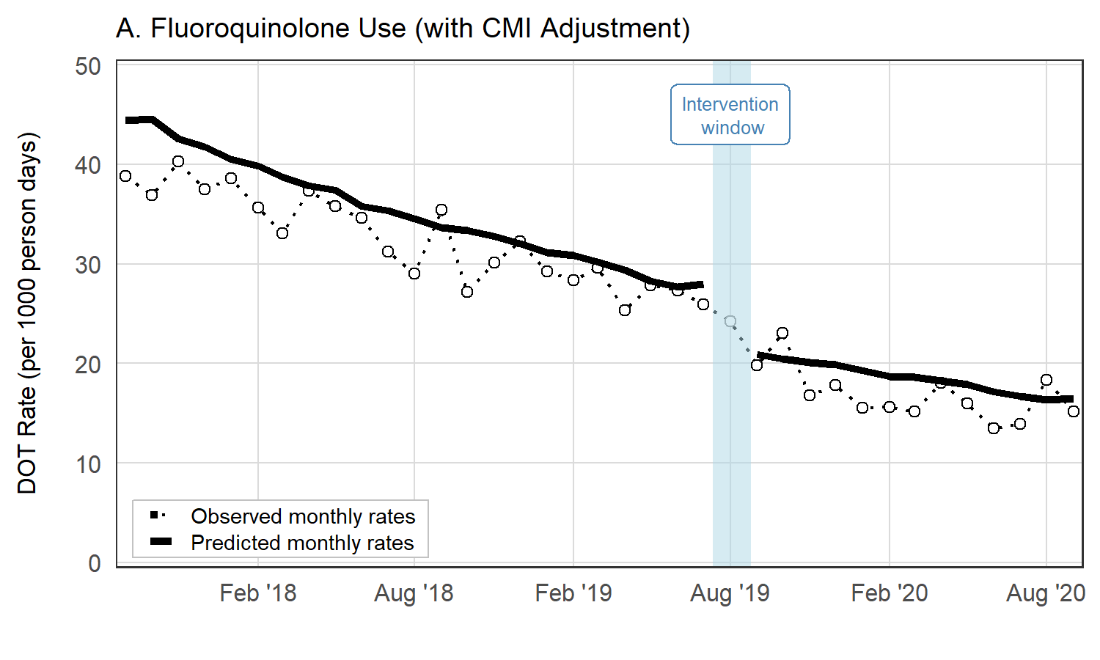

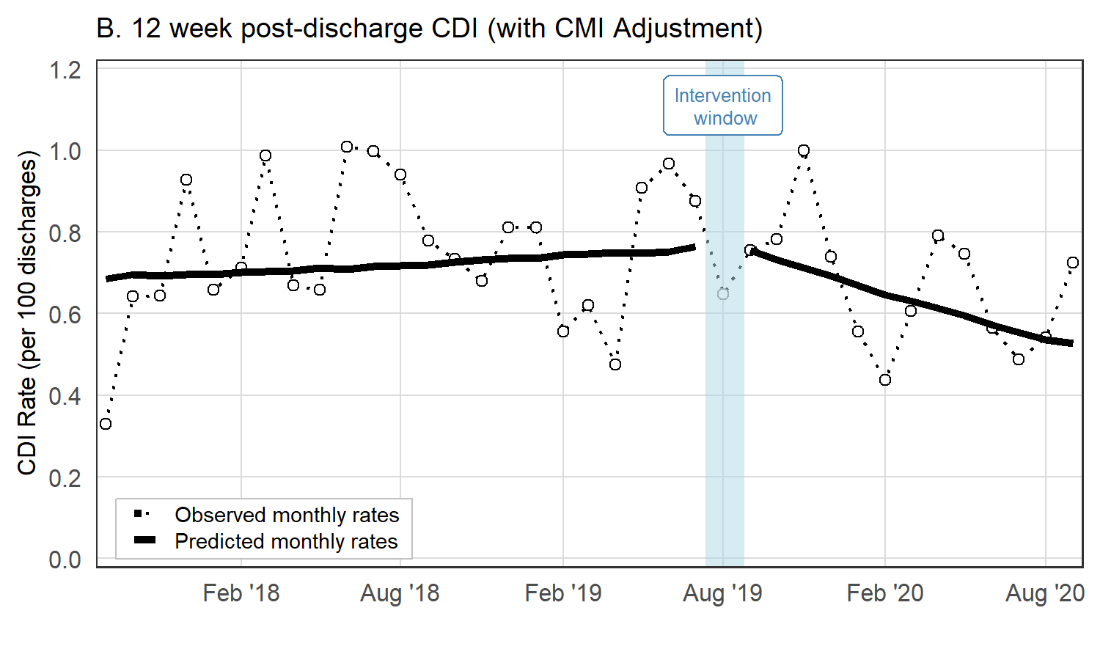


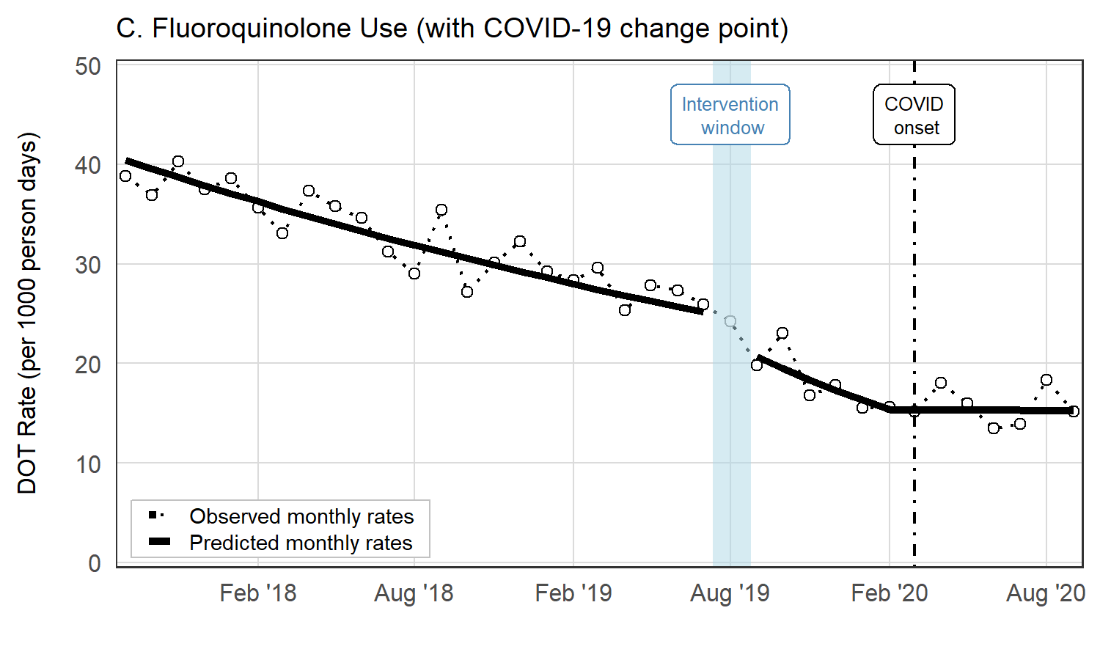

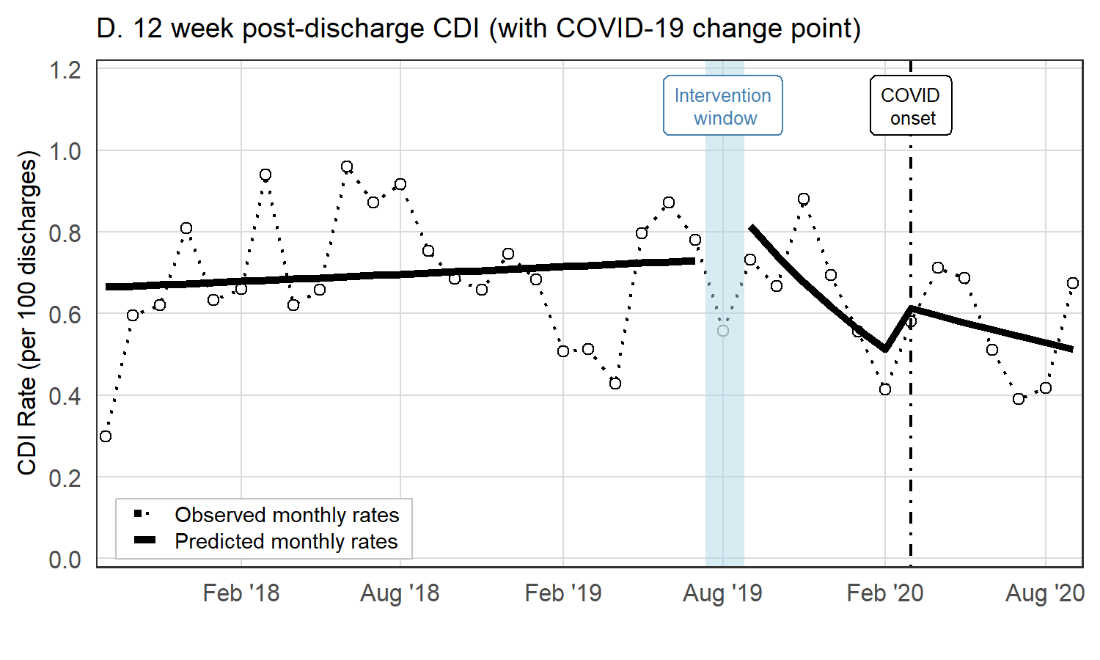


Supplementary Materials: Table S3. Sensitivity Analysis of impact of intervention on fluoroquinolone antibiotic use and 12-week post discharge *C. difficile* infections at four acute-care facilities, September 2017 – September 2020

|  | **Fluoroquinolone Use** | |  | **12-week post-discharge CDI** | |
| --- | --- | --- | --- | --- | --- |
|  | **Rate Ratio (95% CI)** | **P value** |  | **Rate Ratio (95% CI)** | **P value** |
| **Sensitivity analyses using CMI adjustment** |  |  |  |  |  |
| Baseline trend^a^ | 0.978 (0.965, 0.991) | 0.001 |  | 1.008 (0.996, 1.019) | 0.193 |
| Level change after intervention^b^ | 0.787 (0.616, 1.004) | 0.054 |  | 0.967 (0.892, 1.049) | 0.421 |
| Trend change after intervention^c^ | 0.993 (0.978, 1.008) | 0.364 |  | 0.966 (0.942, 0.991) | 0.007 |
| MS-DRG CMI^d^ | 1.316 (1.113, 1.557) | 0.001 |  | 1.100 (0.852, 1.421) | 0.465 |
| Post-intervention trend^e^ | 0.971 (0.960, 0.982) | 0.000 |  | 0.973 (0.959, 0.988) | <0.001 |
| **Sensitivity analyses modeling COVID as a separate change point** |  |  |  |  |  |
| Baseline trend^a^ | 0.979 (0.977, 0.991) | 0.001 |  | 1.008 (0.997, 1.019) | 0.172 |
| Level change after intervention^b^ | 0.872 (0.600, 1.269) | 0.473 |  | 1.192 (0.920, 1.542) | 0.184 |
| Trend change after intervention^c^ | 0.963 (0.903, 1.028) | 0.257 |  | 0.899 (0.793, 1.020) | 0.099 |
| Level change after COVID onset^f^ | 1.058 (0.750, 1.492) | 0.749 |  | 1.335 (0.749, 2.382) | 0.327 |
| Trend change after COVID onset^g^ | 1.060 (0.981, 1.146) | 0.142 |  | 1.089 (0.973, 1.218) | 0.139 |
| Post-intervention trend^e^ | 0.943 (0.880, 1.010) | 0.095 |  | 0.906 (0.800, 1.027) | 0.122 |
| Post-COVID onset trend^h^ | 1.037 (0.963, 1.117) | 0.334 |  | 1.097 (0.978, 1.230) | 0.113 |

^a^Baseline month-to-month trend in rate i.e. exp(β1)

^b^Step change associated with intervention i.e. exp(β2)

^c^Change in month-to-month trend in the post-intervention versus the pre-intervention period i.e. exp(β3)

^d^Medicare Severity Diagnosis Related Group case mix index (MS-DRG)

^e^Month-to-month trend in the post-intervention period only i.e. exp(β1+β3)

^f^Step change associated with COVID onset i.e. exp(β4)

^g^Change in month-to-month trend in the post-COVID-onset versus the pre-intervention periods i.e. exp(β5)

^h^Month-to-month trend in the post-COVID period only i.e. exp(β1+β5)
